# Lifetime history of TBI with loss of consciousness and disability among Appalachian and rural residents: 2016–2019 Ohio BRFSS

**DOI:** 10.1186/s40621-022-00390-w

**Published:** 2022-08-15

**Authors:** Robyn Feiss, John D. Corrigan, Kele Ding, Cynthia L. Beaulieu, Jennifer Bogner, Jingzhen Yang

**Affiliations:** 1 Center for Injury Research and Policy, The Abigail Wexner Research Institute at Nationwide Children’s Hospital, Columbus, OH USA; 2 Division of Rehabilitation Psychology, Department of Physical Medicine and Rehabilitation, The Ohio State University College of Medicine, Columbus, OH USA; 3 School of Health Sciences, Kent State University, Kent, OH USA; 4 Department of Pediatrics, The Ohio State University College of Medicine, Columbus, OH USA

**Keywords:** Appalachian health, Disability, Population health, Rural health, Traumatic brain injury

## Abstract

**Background:**

While lifetime history of traumatic brain injury (TBI) is associated with increased risk of disabilities, little is known about disability and TBI among Appalachian and other rural residents. This study aimed to examine if the relationship between lifetime history of TBI with loss of consciousness (LOC) and disability differs by location of living (Appalachian vs. non-Appalachian; rural vs. urban).

**Methods:**

We obtained data on lifetime history of TBI with LOC, location of living, and six sources of disability (auditory, visual, cognitive, mobility, self-care related, and independent living-related impairments) from the 2016–2019 Ohio Behavioral Risk Factor Surveillance System. We modeled the disability outcomes with Appalachian living (or rural living), lifetime history of TBI with LOC, and their interaction as independent variables.

**Results:**

Of the 16,941 respondents included, 16.9% had a lifetime history of TBI with LOC, 19.5% were Appalachian residents and 22.9% were rural residents. Among Appalachian residents, 56.1% lived in a rural area. Appalachian (ARR = 1.92; 95%CI = 1.71–2.13) and rural residents (ARR = 1.87; 95%CI = 1.69–2.06) who had a lifetime history of TBI with LOC were at greater risk for having any disability compared to non-Appalachian and urban residents without lifetime history of TBI with LOC, respectively.

**Conclusions:**

Appalachian and rural living and lifetime history of TBI with LOC are risk factors for disability. Future research and health policies should address mechanisms for this risk as well as access to healthcare services following a TBI among Appalachian and rural residents.

## Introduction

Children (Leonhard et al. 2015) and adults (Brown et al. 2019; Yue et al. 2020) living in rural areas are at a greater risk for TBI as well as worse health outcomes following TBI (Brown et al. 2019; Tiesman et al. 2007) compared to their urban and suburban counterparts. Furthermore, living in Appalachian and/or rural areas has been associated with various health disparities (Meit et al. 2014; Wewers et al. 2006), including disability (Bouldin et al. 2020; VonReichert et al. 2014; Zhao et al., 2019). Previous studies show that rates of disability were higher among both Appalachian and rural populations compared to non-Appalachian and urban populations, respectively (Bouldin et al. 2020; VonReichert et al. 2014; Zhao et al., 2019). The observed disparities are likely attributed to environmental, cultural, and socioeconomic factors, such as access to quality healthcare and poverty rates, in both Appalachian and/or rural areas (Wewers et al. 2006; Bouldin et al. 2020; Zhao et al., 2019). The Appalachian region of the USA stretches across 13 states and boasts a population of 26 million residents (Appalachian Regional Commission [ARC] 2021; Pollard et al. 2021). In Ohio, the Appalachian region includes 29 of the state’s 88 counties and holds 12% of the state’s population (ARC; Pollard et al. 2021). In comparison with the rest of the state, the Appalachian region has greater rates of poverty and lower rates of health insurance coverage (Pollard et al. 2021), which may contribute to health disparities among these residents. Additionally, there is a large overlap between Appalachian and rural counties in Ohio, with 69% of Appalachian counties in Ohio being considered rural compared to 50% of non-Appalachian counties being considered rural (U.S. Department of Health and Human Services [HHS] et al. 2010). However, little is known about disability and traumatic brain injury (TBI) in Appalachian and rural areas of the USA.

A TBI is a neurological condition resulting from an external force that temporarily or permanently impacts typical brain function (Menon et al. 2010). TBI has been shown to be associated with various short and long-term health consequences, including disability (Sarmiento et al. 2022; Yi et al. 2018). TBI has been strongly linked with cognitive impairments, as well as other sources of disability (Gorgoraptis et al. 2019; Rabinowitz and Levin 2014; Whiteneck et al. 2004). One population-based study found that lifetime history of TBI with loss of consciousness (LOC), regardless of duration of LOC, was associated with greater risk for auditory, visual, cognitive, mobility, self-care related, and independent living-related impairments among adult Ohioans (Yi et al. 2018). However, research has not yet addressed how environmental factors, such as location of living, may be related to the associations between TBI and risk of disability outcomes.

Provided that both lifetime history of TBI, particularly with LOC, and living in an Appalachian or rural area contribute to the risk of disability, this study aims to determine if the association between lifetime history of TBI with LOC and disability differs by location of living (Appalachian vs. non-Appalachian, rural vs. urban). We hypothesized that living in an Appalachian or rural area and having a lifetime history of TBI with LOC would be associated with increased risk of disability. Additionally, the relationship between lifetime history of TBI with LOC and disability may vary based on location of living.

## Methods

All methods were carried out in accordance with the ethical standards as laid down in the 1964 Declaration of Helsinki and its later amendments or comparable ethical standards. The IRB at Nationwide Children’s Hospital determined that this study was not research involving human subjects as defined by DHHS and FDA regulations and does not require IRB review and approval.

### Data and study participants

Data were sourced from the 2016 through 2019 Ohio BRFSS administered to non-institutionalized adults aged 18 years or older (Centers for Disease Control and Prevention [CDC] 2020). The BRFSS is an annual population-based survey which uses random-digit dialing to collect health-related risk behavior and chronic condition-related information via a phone survey (CDC). From 2016 to 2019, the Ohio BRFSS included an optional module addressing lifetime history of TBI via an adapted version of the Ohio State University TBI Identification Method (OSU TBI-ID) (Corrigan and Bogner 2007). Our sample included 19,896 non-institutionalized adults at least 18 years of age who completed both the core component questions and the lifetime history of TBI module of the 2016, 2017, 2018, or 2019 Ohio BRFSS. Of these respondents, 2955 (14.9%) were excluded from the analysis, due to missing location of living information (2.1%, *n* = 410), being less than 18 years old (0.9%, *n* = 173), or unknown history of TBI (11.9%, *n* = 2372), resulting in a final sample of 16,941 respondents. The sample from each year was weighted using iterative proportional fitting or raking methodology to ensure that estimates are representative of the Ohio adult population. For this study, re-weighting was applied to ensure an equal proportion of participation in the data set by year, which multiplied the original sample weight for each year by sample proportion of the year in the combined data set (*n* = 19,896) (BRFSS 2020).

### Variables and measures

Lifetime history of TBI with LOC was defined as an injury to the head or neck that resulted in being knocked out or losing consciousness. The adapted version of the OSU TBI-ID uses 6 items to measure lifetime history of TBI with LOC (Corrigan et al. 2020). Individuals who reported at least one injury to the head or neck were asked if they were ever knocked out or lost consciousness from their reported injury. Responses were used to determine lifetime history of TBI with LOC (yes vs. no). Reliability and validity of the OSU TBI-ID have been established in several studies using a similar data collection method (Corrigan and Bogner 2007; Bogner and Corrigan 2009; Bogner et al. 2017; Cuthbert et al. 2016).

Living in an Appalachian vs. non-Appalachian area was defined based on the ARC’s definition of the Appalachian Region (ARC). Living in a rural area (living in a non-metropolitan or non-micropolitan county in Ohio) vs urban area (living in a metropolitan or micropolitan county in Ohio) was defined based on the Office of Management and Budget (OMB) criteria. Metropolitan counties contain a core urban area of 50,000 or more people and micropolitan counties contain a core urban area of 10,000 to 49,999 people (HHS et al.). Any county not defined as metropolitan or micropolitan was defined as rural as the OMB criteria do not explicitly define rural areas. Counties were identified based on their Federal Information Processing Standards (FIPS) codes.

Six sources of current disability due to impairments are determined by the BRFSS, including auditory, visual, cognitive, mobility, self-care related, and independent living-related impairments (CDC). Current disabilities due to impairments were determined by the following questions: (1) “Are you deaf or do you have serious difficulty hearing?” (auditory); (2) “Are you blind or do you have serious difficulty seeing, even when wearing glasses?” (visual); (3) “Because of a physical, mental, or emotional condition, do you have serious difficulty concentrating, remembering, or making decisions?” (cognitive); (4) “Do you have serious difficulty walking or climbing stairs?” (mobility); (5) “Do you have difficulty dressing or bathing?” (self-care related); and 6) “Because of a physical, mental, or emotional condition, do you have difficulty doing errands alone such as visiting a doctor’s office or shopping?” (independent living related). Respondents who answered “Yes” to any of six questions were categorized as having any disability. The number of disabilities was calculated by summing the number of “yes” responses and then categorized into 4 levels (0, 1, 2, and ≥ 3).

### Statistical analysis

We calculated unweighted and weighted frequencies and percentages of survey respondents along with their demographics. In addition, we calculated weighted frequencies and percentages of survey respondents who reported any and each of auditory, visual, cognitive, mobility, self-care, or independent living-related disabilities. We then compared differences in disability outcomes across subgroups using χ2 tests and used the False Discovery Rate (FDR) method to adjust for multiple comparisons. Finally, we conducted multivariable logistic regressions to determine the relative risk (RR) for each of the disability outcomes (any disability, source of disability, and number of disabilities), with Appalachian living, lifetime history of TBI with LOC, and their interaction as independent variables. Variables adjusted for in the logistic regression models included gender, age group, and race/ethnicity. We repeated the same analysis for rural living. All model tests were also adjusted for sample stratum, cluster, and proportional weight variables. All analyses were performed in SAS 9.4 (SAS Institute, Cary, NC).

## Results

A total of 16,941 survey participants were included, with 7128 from 2016 and 3128, 3328, and 3357 from the 2017, 2018, and 2019 surveys, respectively, due to split assignment of the state-optional module eliciting lifetime TBI with LOC. When weighted, the cohort represented 7,578,787 non-institutionalized adults in Ohio, including 52.1% females, 43.1% aged 18 to 44 years, 81.2% White, 19.5% living in an Appalachian area, and 22.9% living in a rural area (Table 1). The prevalence of experiencing at least one TBI with LOC in their lifetime was 16.9%.

**Table 1:** Demographics and lifetime history of TBI (original and weighted samples)

	Total Population	
	Unweighted *n*	Weighted *n (%)*
Overall	16,941	7,578,787 (100.0)
Survey Year		
2016	7128	3,165,459 (41.8)
2017	3128	1,409,229 (18.6)
2018	3328	1,493,316 (19.7)
2019	3357	1,510,783 (19.9)
Appalachian		
No	11,369	6,102,306 (80.5)
Yes	5572	1,476,482 (19.5)
Rural		
No	10,285	5,841,685 (77.1)
Yes	6656	1,737,102 (22.9)
Gender		
Male	6939	3,631,381 (47.9)
Female	10,002	3,947,407 (52.1)
Age group		
Age 18 to 44	3596	3,262,709 (43.1)
Age 45 to 64	6441	2,613,487 (34.5)
Age 65 or older	6904	1,702,592 (22.5)
Race		
White	14,902	6,150,936 (81.2)
Black	1077	838,535 (11.1)
Other^a^	731	485,575 (6.4)
Unknown	231	103,741 (1.4)
Lifetime history of TBI with LOC		
No	14,213	6,296,117 (83.1)
Yes	2728	1,282,670 (16.9)

Data were expressed as weighted number (*n*) and percent prevalence (%). Totals do not add up to 100% due to rounding. The weighted frequencies and prevalence percentages were estimated using the statewide weighting variable of individuals with the weighted survey procedures in SAS

*TBI* traumatic brain injury, *LOC* loss of consciousness

^a^Other includes Asians, Native Hawaiians, other Pacific Islanders, American Indians, or Alaska Natives

The prevalence rate of any disability among non-institutionalized adults in Ohio was 28.6%, including 7.1% due to auditory impairment, 4.7% due to visual impairment, 12.1% due to cognitive impairment, 15.3% mobility limitations, 3.7% the inability to independently perform self-care, and 7.3% the inability to independently navigate the community (Table 2).

**Table 2:** Associations of lifetime history of TBI and demographics with auditory, visual, cognitive, mobility, self-care, and independent living-related disabilities

	Any disability	Auditory	Visual	Cognitive	Mobility	Self-care	Independent living
	Weighted n (%)	Weighted n (%)	Weighted n (%)	Weighted n (%)	Weighted n (%)	Weighted n (%)	Weighted n (%)
Overall	2,169,504 (28.6)	540,042 (7.1)	353,160 (4.7)	914,067 (12.1)	1,163,190 (15.3)	281,547 (3.7)	554,245 (7.3)
Gender							
Male	1,018,834 (28.1)	315,574 (8.7)***	169,231 (4.7)	430,179 (11.8)	486,195 (13.4)***	140,587 (3.9)	220,390 (6.1)***
Female	1,150,670 (29.2)	224,468 (5.7)	183,929 (4.7)	483,888 (12.3)	676,996 (17.2)	140,961 (3.6)	333,855 (8.5)
Age group							
Age 18 to 44	641,340 (19.7)***	79,833 (2.4)***	97,334 (3.0)***	430,891 (13.2)***	187,181 (5.7)***	81,855 (2.5)***	210,390 (6.4)*
Age 45 to 64	812,754 (31.1)	188,523 (7.2)	143,388 (5.5)	333,772 (12.8)	523,695 (20.0)	124,643 (4.8)	217,747 (8.3)
Age 65 or older	715,409 (42.0)	271,686 (16.0)	112,439 (6.6)	149,404 (8.8)	452,315 (26.6)	75,049 (4.4)	126,108 (7.4)
Race							
White	1,720,284 (28.0)	479,186 (7.8)***	254,874 (4.1)***	688,613 (11.2)***	920,707 (15.0)*	207,184 (3.4)*	417,822 (6.8)**
Black	269,571 (32.1)	24,148 (2.9)	55,023 (6.6)	128,687 (15.3)	155,338 (18.5)	44,780 (5.3)	75,014 (8.9)
Other	148,350 (30.6)	25,689 (5.3)	35,554 (7.3)	85,077 (17.5)	68,774 (14.2)	23,007 (4.7)	54,041 (11.1)
Unknown	31,299 (30.2)	11,019 (10.6)	7709 (7.4)	11,690 (11.3)	18,371 (17.7)	6576 (6.3)	7368 (7.1)
Appalachian							
No	1,665,555 (27.3)	408,699 (6.7)	267,511 (4.4)	694,903 (11.4)	890,244 (14.6)	205,505 (3.4)	413,077 (6.8)
Yes	503,949 (34.1)***	131,343 (8.9)***	85,649 (5.8)**	219,164 (14.8)***	272,946 (18.5)***	76,042 (5.2)***	141,168 (9.6)***
Rural							
No	1,611,629 (27.6)	389,879 (6.7)	260,422 (4.5)	691,239 (11.8)	853,808 (14.6)	213,412 (3.7)	409,101 (7.0)
Yes	557,875 (32.1)***	150,163 (8.6)***	92,738 (5.3)	222,828 (12.8)	309,382 (17.8)***	68,135 (3.9)	145,143 (8.4)*
Lifetime history of TBI with LOC							
No	1,631,251 (25.9)	409,306 (6.5)	264,979 (4.2)	618,867 (9.8)	874,434 (13.9)	182,652 (2.9)	353,516 (5.6)
Yes	538,252 (42.0)***	130,736 (10.2)***	88,181 (6.9)***	295,200 (23.0)***	288,756 (22.5)***	98,896 (7.7)***	200,729 (15.6)***

	Number of disabilities			
	0	1	2	3
Gender				
Male	2,612,547 (71.9)	583,544 (16.1)	237,843 (6.5)	197,447 (5.4)
Female	2,796,737 (70.9)	632,050 (16.0)	265,649 (6.7)	252,971 (6.4)
Age group				
Age 18 to 44	2,621,369 (80.3)	375,764 (11.5)	141,451 (4.3)	124,126 (3.8)***
Age 45 to 64	1,800,732 (68.9)	421,074 (16.1)	183,563 (7.0)	208,118 (8.0)
Age 65 or older	987,182 (58.0)	418,757 (24.6)	178,478 (10.5)	118,174 (6.9)
Race				
White	4,430,652 (72.0)	986,710 (16.0)	389,984 (6.3)	343,590 (5.6)
Black	568,964 (67.9)	144,089 (17.2)	65,733 (7.8)	59,749 (7.1)
Other	337,226 (69.4)	70,530 (14.5)	38,443 (7.9)	39,376 (8.1)
Unknown	72,442 (69.8)	14,265 (13.8)	9,332 (9.0)	7,702 (7.4)
Appalachian				
No	4,436,751 (72.7)	953,087 (15.6)	381,501 (6.3)	330,967 (5.4)
Yes	972,532 (65.9)	262,507 (17.8)	121,991 (8.3)	119,451 (8.1)***
Rural				
No	4,230,057 (72.4)	908,544 (15.6)	367,597 (6.3)	335,487 (5.7)
Yes	1,179,227 (67.9)	307,050 (17.7)	135,894 (7.8)	114,931 (6.6)***
Lifetime history of TBI with LOC				
No	4,664,866 (74.1)	986,002 (15.7)	358,987 (5.7)	286,262 (4.5)
Yes	744,418 (58.0)	229,591 (17.9)	144,505 (11.3)	164,156 (12.8)***

^*^p < .05, **p < .01, ***p < .001, after false discovery rate adjustment for multiple comparisons

*TBI* traumatic brain injury, *LOC* loss of consciousness

Note: Percentages represent the proportion of the subgroup that reported the outcome

Living in an Appalachian area was associated with increased proportions of auditory (*P* < 0.001), visual (*P* = 0.008), cognitive (*P* < 0.001), mobility (*P* < 0.001), self-care (*P* < 0.001), and independent living (*P* < 0.001) sources of disability. Living in a rural area was also associated with increased proportions of auditory (P < 0.001), mobility (*P* < 0.001), and independent living (*P* = 0.04) sources of disability. Additionally, lifetime history of TBI with LOC was associated with increased proportions of auditory (*P* < 0.001), visual (*P* < 0.001), cognitive (*P* < 0.001), mobility (*P* < 0.001), self-care (P < 0.001), and independent living (*P* < 0.001) sources of disability. Finally, the number of disabilities differed by age group (*P* < 0.001), living in an Appalachian area (*P* < 0.001), living in a rural area (*P* < 0.001), and lifetime history of TBI with LOC (*P* < 0.001) (Table 2).

Compared to individuals living in a non-Appalachian region without a lifetime history of TBI with LOC, individuals living in an Appalachian region with a lifetime history of TBI with LOC were at increased risk for having any disability (ARR = 1.92; 95%CI = 1.71–2.13), after adjusting for gender, age group, and race (Table 3). Increased risk for having a disability was also observed among individuals living in non-Appalachian regions with a lifetime history of TBI with LOC (ARR = 1.73; 95%CI = 1.57–1.88) and individuals living in an Appalachian region without a lifetime history of TBI with LOC (ARR = 1.28; 95%CI = 1.18–1.39). Further, individuals living in an Appalachian region with a lifetime history of TBI with LOC were at increased risk for having each of the six individual sources of disability as compared to those living in a non-Appalachian area without a lifetime history of TBI with LOC. Similar findings were observed among individuals either living in an Appalachian region or with a lifetime history of TBI with LOC. Finally, those living in an Appalachian region with a lifetime history of TBI with LOC were at greater risk for reporting two (ARR = 2.40; 95%CI = 1.78–3.02), and three or more sources of disability (AAR = 4.25; 95%CI = 3.05–5.44) compared to those in non-Appalachian areas without a lifetime history of TBI with LOC. Increased risk for reporting one, two, or three disabilities was also observed among individuals either living in an Appalachian region or with a lifetime history of TBI with LOC.

**Table 3:** Adjusted relative risk ratios (RR) for Appalachian vs non-Appalachian residents for any disability, sources of disability, and number of disabilities

	Any disability			Auditory			Visual			Cognitive		
	Adjusted RR	95% CI		Adjusted RR	95% CI		Adjusted RR	95% CI		Adjusted RR	95% CI	
Lifetime history of TBI with LOC*Appalachian												
Yes, Appalachian	**1.92**	**1.71**	**2.13**	**2.10**	**1.57**	**2.63**	**2.28**	**1.59**	**2.98**	**2.88**	**2.30**	**3.45**
Yes, Non-Appalachian	**1.73**	**1.57**	**1.88**	**1.73**	**1.35**	**2.11**	**1.74**	**1.28**	**2.20**	**2.43**	**2.04**	**2.82**
No, Appalachian	**1.28**	**1.18**	**1.39**	**1.28**	**1.06**	**1.50**	**1.39**	**1.06**	**1.72**	**1.41**	**1.17**	**1.65**
No, Non-Appalachian	Ref	Ref	Ref	Ref	Ref	Ref	Ref	Ref	Ref	Ref	Ref	Ref
Gender												
Male	0.96	0.90	1.02	**1.62**	**1.38**	**1.86**	0.99	0.80	1.18	0.89	0.78	1.01
Female	Ref	Ref	Ref	Ref	Ref	Ref	Ref	Ref	Ref	Ref	Ref	Ref
Race												
Non-White	**1.25**	**1.15**	**1.36**	**0.69**	**0.50**	**0.87**	**1.94**	**1.49**	**2.38**	**1.45**	**1.22**	**1.68**
White	Ref	Ref	Ref	Ref	Ref	Ref	Ref	Ref	Ref	Ref	Ref	Ref
Age group												
Age 18 to 44	**0.49**	**0.45**	**0.53**	**0.15**	**0.11**	**0.19**	**0.41**	**0.30**	**0.52**	**1.35**	**1.15**	**1.56**
Age 45 to 64	**0.75**	**0.71**	**0.79**	**0.43**	**0.37**	**0.49**	**0.79**	**0.64**	**0.93**	**1.33**	**1.14**	**1.51**
Age 65 +	Ref	Ref	Ref	Ref	Ref	Ref	Ref	Ref	Ref	Ref	Ref	Ref

	Mobility			Self-care			Independent living		
	Adjusted RR	95% CI		Adjusted RR	95% CI		Adjusted RR	95% CI	
Lifetime history of TBI with LOC* Appalachian									
Yes, Appalachian	**2.12**	**1.77**	**2.47**	**4.21**	**2.47**	**5.95**	**3.90**	**2.88**	**4.92**
Yes, Non-Appalachian	**1.91**	**1.64**	**2.17**	**2.72**	**1.90**	**3.53**	**3.08**	**2.45**	**3.71**
No, Appalachian	**1.30**	**1.15**	**1.45**	**1.56**	**1.15**	**1.98**	**1.50**	**1.20**	**1.81**
No, Non-Appalachian	Ref	Ref	Ref	Ref	Ref	Ref	Ref	Ref	Ref
Gender									
Male	**0.78**	**0.70**	**0.86**	1.01	0.79	1.23	**0.66**	**0.55**	**0.77**
Female	Ref	Ref	Ref	Ref	Ref	Ref	Ref	Ref	Ref
Race									
Non-White	**1.43**	**1.25**	**1.61**	**1.83**	**1.32**	**2.33**	**1.56**	**1.23**	**1.89**
White	Ref	Ref	Ref	Ref	Ref	Ref	Ref	Ref	Ref
Age group									
Age 18 to 44	**0.22**	**0.18**	**0.26**	**0.50**	**0.35**	**0.65**	**0.79**	**0.64**	**0.95**
Age 45 to 64	**0.74**	**0.69**	**0.80**	0.97	0.77	1.17	1.03	0.87	1.19
Age 65 +	Ref	Ref	Ref	Ref	Ref	Ref	Ref	Ref	Ref

	1 Disability			2 Disabilities			3 + Disabilities		
	Adjusted RR	95% CI		Adjusted RR	95% CI		Adjusted RR	95% CI	
Lifetime history of TBI with LOC*Appalachian									
Yes, Appalachian	1.11	0.88	1.33	**2.40**	**1.79**	**3.02**	**4.25**	**3.05**	**5.44**
Yes, Non-Appalachian	**1.23**	**1.05**	**1.41**	**2.22**	**1.71**	**2.73**	**2.92**	**2.27**	**3.57**
No, Appalachian	**1.18**	**1.03**	**1.32**	**1.43**	**1.13**	**1.72**	**1.49**	**1.18**	**1.79**
No, Non-Appalachian	Ref	Ref	Ref	Ref	Ref	Ref	Ref	Ref	Ref
Gender									
Male	1.04	0.93	1.14	0.95	0.79	1.12	**0.79**	**0.65**	**0.93**
Female	Ref	Ref	Ref	Ref	Ref	Ref	Ref	Ref	Ref
Race									
Non-White	1.07	0.92	1.22	**1.44**	**1.11**	**1.77**	**1.57**	**1.23**	**1.91**
White	Ref	Ref	Ref	Ref	Ref	Ref	Ref	Ref	Ref
Age group									
Age 18 to 44	**0.51**	**0.45**	**0.58**	**0.41**	**0.31**	**0.51**	**0.52**	**0.40**	**0.65**
Age 45 to 64	**0.68**	**0.61**	**0.75**	**0.65**	**0.55**	**0.75**	1.08	0.90	1.26
Age 65 +	Ref	Ref	Ref	Ref	Ref	Ref	Ref	Ref	Ref

Adjusted for gender, age group, race, and Appalachian by lifetime history of TBI with LOC interaction

*TBI* traumatic brain injury, *LOC* loss of consciousness

Bolded values represent statistically significant values (*p* < .05)

After adjusting for gender, age group, and race, individuals living in a rural area with a lifetime history of TBI with LOC were at increased risk for having any disability (ARR = 1.87; 95%CI = 1.69–2.06) compared to individuals without a lifetime history of TBI with LOC living in an urban area (Table 4). Furthermore, compared to individuals living in an urban area without a lifetime history of TBI with LOC, those living in an urban area with a lifetime history of TBI with LOC as well as those living in a rural area without a lifetime history of TBI with LOC were also at increased risk for having any disability (ARR = 1.18; 95%CI = 1.09–1.28). Additionally, those with a lifetime history of TBI with LOC living in rural areas were at increased risk for reporting one (ARR = 1.25; 95%CI = 1.02–1.48), two (ARR = 2.66; 95%CI = 2.00–3.32), and 3 or more (ARR = 3.10; 95%CI = 2.30–3.90) sources of disability, compared to those living in urban areas without a lifetime history of TBI with LOC. Greater risk for reporting one, two, or three disabilities was also observed among individuals either living in a rural area or with a lifetime history of TBI with LOC, although the relative risk for reporting two disabilities was not statistically significant for those living in a rural area without a lifetime history of TBI with LOC.

**Table 4:** Adjusted relative risk ratios (RR) for rural vs urban residents for any disability, sources of disability, and number of disabilities

	Any disability			Auditory			Visual			Cognitive		
	Adjusted RR	95% CI		Adjusted RR	95% CI		Adjusted RR	95% CI		Adjusted RR	95% CI	
Lifetime history of TBI with LOC*rural												
Yes, Rural	**1.87**	**1.69**	**2.06**	**2.11**	**1.56**	**2.66**	**1.96**	**1.33**	**2.59**	**2.68**	**2.20**	**3.17**
Yes, Urban	**1.70**	**1.55**	**1.86**	**1.68**	**1.30**	**2.06**	**1.83**	**1.35**	**2.31**	**2.33**	**1.94**	**2.71**
No, Rural	**1.18**	**1.09**	**1.28**	1.18	0.99	1.38	**1.35**	**1.04**	**1.67**	1.13	0.95	1.30
No, Urban	Ref	Ref	Ref	Ref	Ref	Ref	Ref	Ref	Ref	Ref	Ref	Ref
Gender												
Male	0.96	0.90	1.02	**1.62**	**1.38**	**1.87**	0.99	0.79	1.18	0.89	0.77	1.00
Female	Ref	Ref	Ref	Ref	Ref	Ref	Ref	Ref	Ref	Ref	Ref	Ref
Race												
Non-White	**1.26**	**1.15**	**1.36**	**0.69**	**0.50**	**0.88**	**1.96**	**1.50**	**2.41**	**1.43**	**1.20**	**1.66**
White	Ref	Ref	Ref	Ref	Ref	Ref	Ref	Ref	Ref	Ref	Ref	Ref
Age group												
Age 18 to 44	**0.49**	**0.45**	**0.53**	**0.15**	**0.11**	**0.19**	**0.41**	**0.30**	**0.52**	**1.36**	**1.15**	**1.56**
Age 45 to 64	**0.75**	**0.71**	**0.79**	**0.43**	**0.37**	**0.49**	**0.79**	**0.64**	**0.93**	**1.33**	**1.15**	**1.51**
Age 65 +	Ref	Ref	Ref	Ref	Ref	Ref	Ref	Ref	Ref	Ref	Ref	Ref

	Mobility			Self-care			Independent living		
	Adjusted RR	95% CI		Adjusted RR	95% CI		Adjusted RR	95% CI	
Lifetime history of TBI with LOC*rural									
Yes, Rural	**2.12**	**1.78**	**2.46**	**2.70**	**1.79**	**3.61**	**3.26**	**2.49**	**4.02**
Yes, Urban	**1.89**	**1.62**	**2.16**	**2.94**	**2.05**	**3.84**	**3.19**	**2.52**	**3.85**
No, Rural	**1.24**	**1.10**	**1.38**	1.25	0.94	1.56	**1.36**	**1.09**	**1.63**
No, Urban	Ref	Ref	Ref	Ref	Ref	Ref	Ref	Ref	Ref
Gender									
Male	**0.78**	**0.70**	**0.86**	1.00	0.78	1.23	**0.66**	**0.55**	**0.76**
Female	Ref	Ref	Ref	Ref	Ref	Ref	Ref	Ref	Ref
Race									
Non-White	**1.44**	**1.25**	**1.63**	**1.79**	**1.28**	**2.29**	**1.56**	**1.22**	**1.90**
White	Ref	Ref	Ref	Ref	Ref	Ref	Ref	Ref	Ref
Age group									
Age 18 to 44	**0.22**	**0.18**	**0.26**	**0.50**	**0.35**	**0.64**	**0.79**	**0.63**	**0.95**
Age 45 to 64	**0.75**	**0.69**	**0.80**	0.97	0.77	1.18	1.03	0.87	1.19
Age 65 +	Ref	Ref	Ref	Ref	Ref	Ref	Ref	Ref	Ref

	1 Disability			2 Disabilities			3 + Disabilities		
	Adjusted RR	95% CI		Adjusted RR	95% CI		Adjusted RR	95% CI	
Lifetime history of TBI with LOC*rural									
Yes, Rural	**1.25**	**1.02**	**1.48**	**2.66**	**2.00**	**3.32**	**3.10**	**2.30**	**3.90**
Yes, Urban	**1.18**	**1.00**	**1.36**	**2.05**	**1.56**	**2.55**	**3.11**	**2.41**	**3.80**
No, Rural	**1.14**	**1.01**	**1.26**	1.24	0.99	1.50	**1.27**	**1.02**	**1.53**
No, Urban	Ref	Ref	Ref	Ref	Ref	Ref	Ref	Ref	Ref
Gender									
Male	1.03	0.93	1.14	0.95	0.79	1.12	**0.78**	**0.65**	**0.92**
Female	Ref	Ref	Ref	Ref	Ref	Ref	Ref	Ref	Ref
Race									
Non-White	1.08	0.92	1.23	**1.45**	**1.12**	**1.79**	**1.55**	**1.20**	**1.89**
White	Ref	Ref	Ref	Ref	Ref	Ref	Ref	Ref	Ref
Age group									
Age 18 to 44	**0.51**	**0.45**	**0.58**	**0.41**	**0.31**	**0.51**	**0.52**	**0.40**	**0.65**
Age 45 to 64	**0.68**	**0.62**	**0.75**	**0.65**	**0.55**	**0.76**	1.08	0.90	1.26
Age 65 +	Ref	Ref	Ref	Ref	Ref	Ref	Ref	Ref	Ref

Adjusted for gender, age group, race, and rural by lifetime history of TBI with LOC interaction

*TBI* traumatic brain injury, *LOC* loss of consciousness

Bolded values represent statistically significant values (*p* < .05)

## Discussion

The present study was designed to examine if the relationship between lifetime history of TBI with LOC and disability differs by location of living (Appalachian vs. non-Appalachian and/or rural vs. urban). The main finding showed that living in an Appalachian or rural area and/or having lifetime history of TBI with LOC is associated with increased risk for reporting at least one type of impairment leading to disability compared to those living in a non-Appalachian or urban area and having no lifetime history of TBI with LOC, respectively. However, the interaction of these two factors did not further increase the risk for any of the disability outcomes as indicated by overlapping confidence intervals. Both living in an Appalachian area and having lifetime history of TBI with LOC were associated with increased risk for all six sources of disability, while living in a rural area was only associated with an increased risk for visual, mobility, and independent living impairments. Taken together, our findings suggest that living in Appalachian and/or rural areas and having a lifetime history of TBI with LOC are two important risk factors for disability. Our findings suggest that future interventions targeting reducing and/or managing disabilities due to TBI need to consider location of living and be designed to address the specific needs of Appalachian and/or rural residents.

This is the first study to address the possible influence of living in an Appalachian and/or rural area on the relationship between lifetime history of TBI with LOC and disability. While living in an Appalachian and/or rural area and lifetime history of TBI with LOC both contributed to increased risk of disabilities, lifetime history of TBI with LOC had a greater contribution to the majority of the disability outcomes. This was demonstrated by the greater RRs among the Appalachian and rural groups with a lifetime history of TBI with LOC and non-overlapping confidence intervals between these groups, compared to the Appalachian and rural groups without a lifetime history of TBI with LOC, respectively. These findings are consistent with previous evidence that lifetime history of TBI with LOC is associated with greater risk of all six sources of disability (Sarmiento et al. 2022; Yi et al. 2018). It is possible that factors outside the scope of this study, such as migration and health care utilization, may impact this relationship. Future research addressing TBI and health disparities, including disability, in Appalachian populations should address both migration and health care utilization.

Living in an Appalachian area, regardless of lifetime history of TBI with LOC, was associated with increased risk for any disability as well as all six sources of disability. This finding is consistent with previous research demonstrating health disparities in the Appalachian population. For example, a population-based study using the 2013–2016 North Carolina BRFSS data found that prevalence of all six sources of disability was higher among Appalachian vs non-Appalachian residents (Bouldin et al. 2020). Additionally, the current study found living in an Appalachian area was associated with reporting multiple disabilities. These relationships were maintained after adjusting for gender, race, and age group, suggesting that some aspect(s) of the Appalachian environment, culture, or lifestyle (e.g., greater rates of poverty and decreased access to healthcare) may contribute to these disparities (Wewers et al. 2006; Bouldin et al. 2020; Pollard et al. 2021). Indeed, the World Health Organization suggests that personal and environmental factors, including health conditions, social structure, and available support systems, interact to impact disability (World Health Organization 2002). Given that Appalachian regions, including those in Ohio, are typically associated with lower socioeconomic status (ARC 2021; Pollard et al. 2021), it is likely that income significantly contributes to this disparity (Bouldin et al. 2020). However, further research is needed to determine the direct causes of increased risk of disability among those with a lifetime history of TBI.

Living in a rural area, regardless of lifetime history of TBI with LOC, was associated with increased risk for having any disability and a visual, mobility, or independent living-related issue leading to disability. Consistent with our findings, a recent study using the 2016 National BRFSS data found that those living in rural areas reported higher rates of disability than those in urban areas, including visual, mobility, and independent living-related disabilities (Zhao et al. 2019). While the study also found this difference among cognitive, auditory, and self-care, our study did not. The inconsistent findings may be due to the operationalization of rurality, as our study used a dichotomous definition (rural vs. urban) while the previous study used a more complex definition with 6 different categories (Zhao et al. 2019). Future research addressing the impact of rurality on relationships between lifetime history of TBI and disability may consider using different definitions of rurality to address this and other similar research questions, as evidence has shown that the definition of rurality can impact observed relationships (Hawley et al. 2016; Isserman 2016; Owen et al. 2007).

The findings from the present study suggest that living in an Appalachian area may have further health disadvantages than living in a rural area. Those living in Appalachian areas of Ohio have lower income and are less likely to have health insurance coverage than those living in non-Appalachian areas of Ohio (Pollard et al. 2021). Given that the largest differences in disability are observed between large metropolitan and the most remote rural areas (Zhao et al. 2019), it is possible that greater lack of access to resources may contribute to the additional disparity among Appalachian populations.

### Limitations

The use of a cross-sectional, retrospective design and lack of knowledge regarding disability status prior to TBI prevents the study from addressing the causal relationships between location of living, lifetime history of TBI with LOC, and disability. Longitudinal, prospective study designs should be used in future research to address these limitations. A second limitation was the use of self-report data. Though measures used for lifetime history of TBI and disability have been well validated, as with any self-report, it is possible that recall bias or social desirability may have impacted our findings. Finally, our study did not measure availability of, access to, or utilization of health care services, which are likely impacted by location of living and may be relevant to the study findings. Future studies should consider the availability, accessibility, and utilization of health care among those with lifetime history of TBI and how these factors may influence the relationships discussed in this study.

## Conclusions

This study found that lifetime history of TBI with LOC and living in an Appalachian and/or rural area are all risk factors for disability. Particularly, having a lifetime history of TBI with LOC and living in an Appalachian area were associated with increased risk of all six sources of disability compared to no lifetime history of TBI with LOC or living in a non-Appalachian area. Our findings suggest that future interventions aimed at the reduction and/or management of disabilities due to TBI must be designed to address the specific needs of Appalachian and/or rural residents. Moreover, given those living in Appalachian and/or rural areas may have limited access to quality health care compared to other areas, future research and health policies should address access to healthcare services following a TBI among Appalachian and rural residents.

## Data Availability

The data that support the findings of this study are available from the Ohio Department of Public Health, but restrictions apply to the availability of these data, which were used under license for the current study and, therefore, are not publicly available. Data are, however, available from the authors upon reasonable request and with permission of the Ohio Department of Public Health.

## Abbreviations

ARC: Appalachian Regional Commission
HHS: U.S. Department of Health and Human Services
TBI: Traumatic brain injury
LOC: Loss of consciousness
BRFSS: Behavioral risk factor surveillance system
CDC: Centers for Disease Control and Prevention
FIPS: Federal Information Processing Standards
